# Effect of Sunlight on the Change in Color of Unsteamed and Steamed Beech Wood with Water Steam

**DOI:** 10.3390/polym14091697

**Published:** 2022-04-21

**Authors:** Michal Dudiak, Ladislav Dzurenda, Viera Kučerová

**Affiliations:** Faculty of Wood Sciences and Technology, Technical University in Zvolen, T.G. Masaryka 24, 96001 Zvolen, Slovakia; dzurenda@tuzvo.sk (L.D.); viera.kucerova@tuzvo.sk (V.K.)

**Keywords:** beech wood, thermal treatment, saturated water steam, natural aging, wood color, ATR-FTIR spectroscopy

## Abstract

This paper presents the differences in the color changes of unsteamed and steamed beech wood (*Fagus sylvatica* L.) caused by long-term exposure to sunlight on the surface of wood in interiors for 36 months. The light white-gray color of the yellow tinge of native beech wood darkened under the influence of sunlight, and the wood took on a pale brown color of yellow tinge. The degree of darkening and browning is quantified by the value of the total color difference ∆E* = 13.0. The deep brown-red color of steamed beech under the influence of sunlight during the exposure brightened, and the surface of the wood took on a pale brown hue. The degree of lightening of the color of steamed beech wood in the color space CIE L*a*b* is quantified by the value of the total color difference ∆E* = 7.1. A comparison of the color changes of unsteamed and steamed beech wood through the total color difference ∆E* due to daylight shows that the surface of steamed beech wood shows 52.2% smaller changes than unsteamed beech wood. The lower value of the total color difference of steamed beech wood indicates the fact that steaming of beech wood with saturated water steam has a positive effect on the color stability and partial resistance of steamed beech wood to the initiation of photochemical reactions induced by UV–VIS wavelengths of solar radiation. Spectra ATR-FTIR analyses declare the influence of UV–VIS components of solar radiation on unsteamed and steamed beech wood and confirm the higher color stability of steamed beech wood.

## 1. Introduction

The color of wood is a basic physical–optical property, which belongs to the group of macroscopic features on the basis of which the wood of individual woody plants differs visually. The color of the wood is formed by chromophores, i.e., functional groups of the type >C=O, -CH=CH-CH=CH-, -CH=CH-, aromatic nuclei found in the chemical components of wood (lignin and extractive substances such as dyes, tannins, resins and others), which absorb some components of the electromagnetic radiation of daylight and thus create the color of the wood surface perceived by human vision.

Using the coordinates of the color space CIE L*a*b* is one of the ways to quantify the given optical wood property objectively. Lab color space (according to CIE-Commission Internationale d’Eclairage) in accordance with ISO 11 664-4 is based on the measurement of three parameters: brightness L* represents the darkest black at L* = 0 and the brightest white at L* = 100. The value of a* is a measure of the red-green character of the color, with positive values for red shades (+a*), and negative values for green (−a*). The value of b* gives the yellow-blue character with positive values for yellow shades (+b*) and negative for blue (−b*).

Wood with long-term exposure to sunlight changes color on its surface. The surface of the wood darkens and mostly yellows and browns. This fact is also referred to in the professional literature as natural aging [1,2,3].

Solar radiation is electromagnetic radiation with wavelengths in the range from 100 to 3000 nm [4], which consists of ultraviolet radiation, visible radiation (light) and infrared radiation. Ultraviolet radiation (UV) with wavelengths of 100–380 nm makes up about 2% of the daylight spectrum. According to the effect of UV radiation on biological materials and their effects on these materials, UV radiation is divided into: UV-A radiation, with a wavelength of 320–380 nm; UV-B radiation, with a wavelength of 280–320 nm; and UV-C radiation, with a wavelength below 280 nm. The spectrum of UV radiation falls on the Earth’s surface from solar radiation, which is made up of 90–99% UV-A radiation and 1–10% UV-B radiation. The most dangerous UV-C radiation is completely absorbed by the atmosphere. The visible light spectrum, referred to as VIS, with wavelengths from 380 to 780 nm, represents approximately 49% of the daylight spectrum. The rest consists of infrared IR radiation with wavelengths of 780–3000 nm. The wavelengths of visible and infrared radiation are absorbed or reflected by the wood surface. The reflected wavelengths of the visible spectrum allow a person to perceive its color when looking at a given object. The absorbed wavelengths of infrared solar radiation change to heat on the surface.

UV–VIS components of solar radiation (daylight) initiate wood photodegradation processes when impacting on the wood surface (photolytic and photo-oxidation reactions with lignin, polysaccharides and wood accessory substances), and carbohydrates absorb 5–20% and 2% of the accessory substance [5]. These reactions cleave both lignin macromolecules with the simultaneous formation of phenolic hydroperoxides, free radicals, carbonyl and carboxyl groups, as well as polysaccharides into polysaccharides, with a lower degree of polymerization to form carbonyl, carboxyl groups and gaseous products (CO, CO_2_, H_2_) [1,3,6,7,8].

The aim of this work is to compare the effect of solar radiation on the surface of thermally treated beech wood with saturated steam (steaming) and unsteamed beech wood. Through changes in the coordinates L*, a*, b* in the color space CIE L*a*b* and the total color difference ∆E*, color changes of native and steamed beech wood caused by UV–VIS components of sunlight (daylight) are evaluated.

## 2. Material and Methods

### 2.1. Material and Technology of Beech Wood Steaming

**Figure 1:**
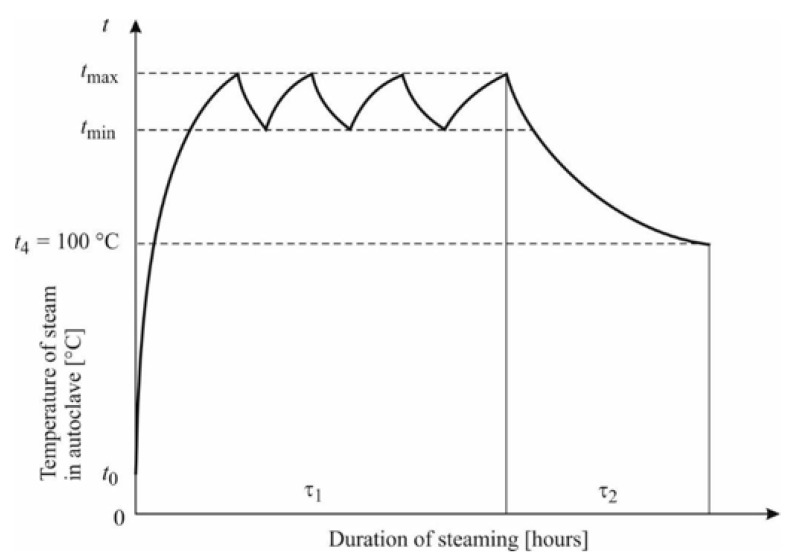
Mode of color modification of beech wood with saturated water steam.

Blanks with dimensions 32 × 60 × 600 mm made of beech wood had a moisture content *w_p_* = 56.4 ± 4.2% and were divided into 2 groups. The blanks of the first group were not thermally steamed prior to drying. The blanks of the second group were steamed to modify the color of the beech wood. Steaming was performed in an APDZ 240 pressure autoclave (Himmasch AD, Haskovo, Bulgaria) installed at Sundermann Ltd. (Banská Štiavnica, Slovakia). The steaming mode of beech wood with saturated water steam is shown in Figure 1, and the technological parameters of the steaming mode are given in Table 1.

### 2.2. Conditions of Beech Wood Exposure

Unsteamed and steamed beech blanks were dried by a low-temperature drying mode preserving the original wood color to a moisture content *w_k_* = 12 ± 0.5% in a convection hot air dryer: KC 1/50 (SUZAR Ltd., Považany, Slovakia) [9].

Samples of the following dimensions were produced from native and steamed beech wood blanks: 20 × 50 × 400 mm. The planed surface of unsteamed and steamed beech wood samples was exposed to daylight for a long time at an angle of 45° in the northern temperate zone (Slovakia, Central Europe) locality for 36 months. The temperature and relative humidity of the indoor air during the exposure were *t* = 20 ± 2.5 °C and φ = 50 ± 10%.

The average density of incident solar radiation in Slovakia is 1100 kWh/m^2^ per year. The intensity of the sun’s rays changes throughout the year. The highest intensity of solar radiation is in the summer months of June and July when it reaches a value of 5.9 to 6.0 kWh/m^2^ per day. During the autumn, the intensity of sunlight decreases and is lowest during the winter. In December, the intensity of solar radiation is the weakest, with an approximate value of 1.7 kWh/m^2^ per day.

### 2.3. Color Measurement of Beech Wood

The surface color of beech samples before and during the exposure was evaluated in the color space CIE L*a*b* at monthly intervals using the Color Reader CR-10 (Konica Minolta, Osaka, Japan) colorimeter was measured. A D65 light source was used, and the diameter of the optical scanning aperture was 8 mm [10].

The total color difference ΔE* of the beech wood surface change during the 36-month exposure to sunlight was determined according to the following ISO 11 664-4 equation:

(1) $$\Delta E^{*}=\sqrt{{\left(L_{2}^{*}-L_{1}^{*}\right)}^{2}+{\left(a_{2}^{*}-a_{1}^{*}\right)}^{2}+{\left(b_{2}^{*}-b_{1}^{*}\right)}^{2}}$$

where $L_{1}^{*}$, $a_{1}^{*}$, $b_{1}^{*}$ are the coordinates of the color space CIE L*a*b* on the surface of the dried, milled beech wood before exposure, and $L_{2}^{*}$, $a_{2}^{*}$, $b_{2}^{*}$ are the coordinates of the color space CIE L*a*b* on the surface of the dried, milled beech wood during exposure.

### 2.4. Mathematical-Statistical Evaluation of Measured Data

The measured values on the brightness coordinate L* and the chromaticity coordinates a*, b*, as well as the calculated values of the total color differences ΔE* during the observed exposure periods, were statistically and graphically evaluated using Excel and Statistica v.12 programs (V12.0 SP2, Palo Alto, CA, USA).

### 2.5. Analysis of Changes in Lignin-Cellulose Matrix of Wood ATR-FTIR Spectroscopy

Fourier-transform infrared spectroscopy (FTIR) was used to follow chemical changes in beech wood after radiation in unsteamed and steamed beech wood. The measurements were carried out using a Nicolet iS10 spectrometer (Thermo Fisher Scientific, Madison, WI, USA) equipped with the Smart iTR ATR accessory.

The spectra were collected in an absorbance mode between 4000 and 650 cm^−1^ by accumulating 32 scans at a resolution of 4 cm^−1^ using diamond crystal. All analyses were performed in four replicates. The spectra were evaluated using the OMNIC 8.0 software.

## 3. Results and Discussion

Beech wood, according to [11,12,13], has a light white-brown-yellow color. In the steaming process with a steam–air mixture at atmospheric pressure, or thermal treatment of beech wood with saturated water steam, as reported by [13,14,15,16], depending on the temperature and length of the thermal treatment process, the wood darkens and acquires shades from a pale pink-yellow color shade to brown-red color. Table 2 shows the coordinates of the color space CIE L*a*b* of unsteamed and steamed beech wood at a moisture content of *w* = 12% on the planed surface before and after 36 months of dazzling. The values for the brightness coordinates L* and the chromatic coordinates a* and yellow b* of the color space CIE L*a*b* of unsteamed beech wood given in Table 1 are similar to those reported by [13,17,18].

**Figure 2:**
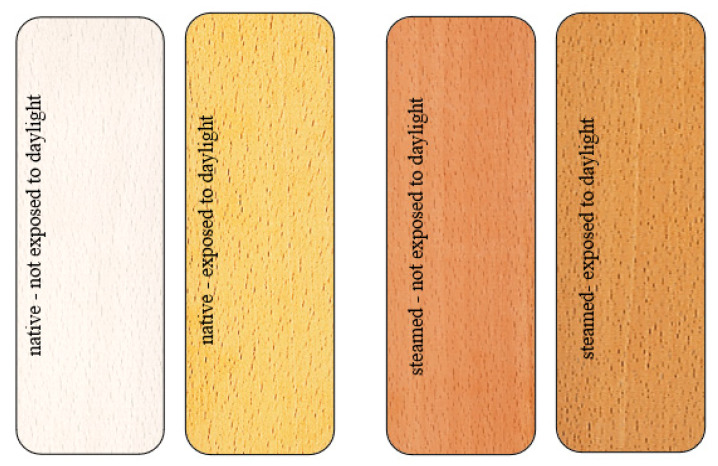
View of beech wood: native before and after exposure (**left**), steamed before and after exposure (**right**).

The color of native and steamed beech wood before and after exposure to daylight glare is shown in Figure 2.

**Figure 3:**
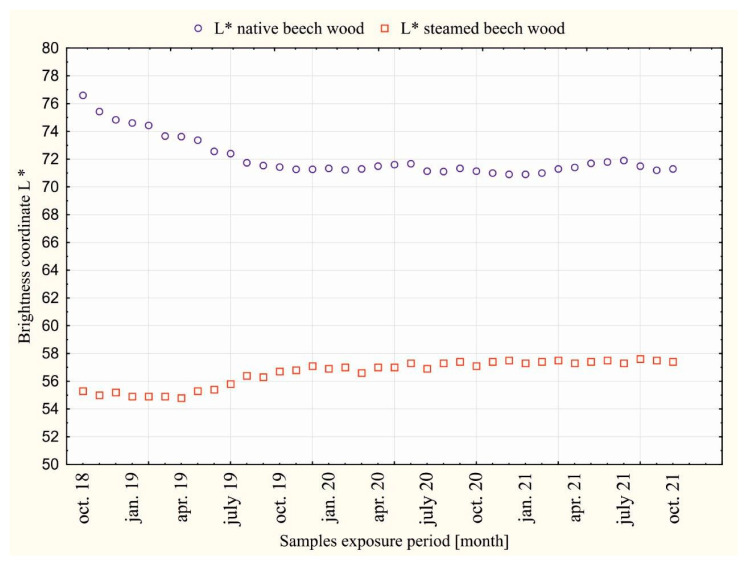
Values on the L* coordinate of dazzled native and steamed beech wood over a period of 36 months (October 2019 to October 2021).

**Figure 4:**
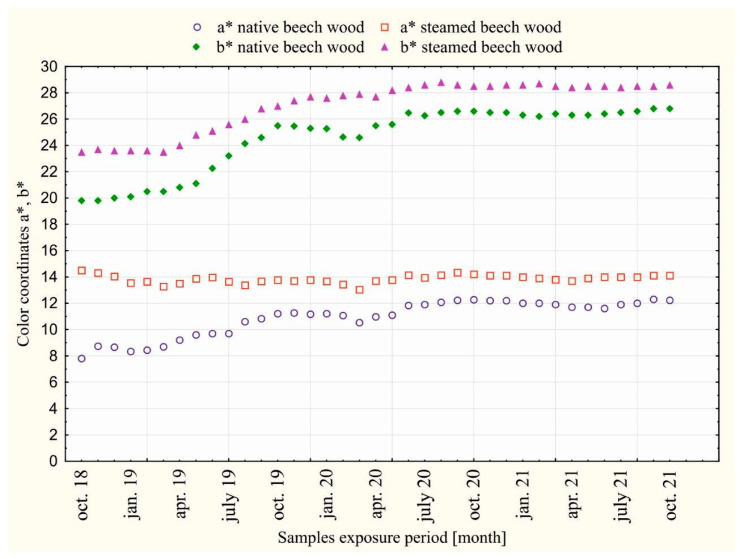
Values on chromatic coordinates of red color a* and yellow color b* of dazzling native and steamed beech wood over a period of 36 months (October 2019 to October 2021).

The courses of the measured values of beech wood color on the coordinates: L*, a*, b* of the color space CIE L*a*b* in individual months, during 36 months of dazzling by the sunlight of daylight are shown in Figure 3 and Figure 4.

**Figure 5:**
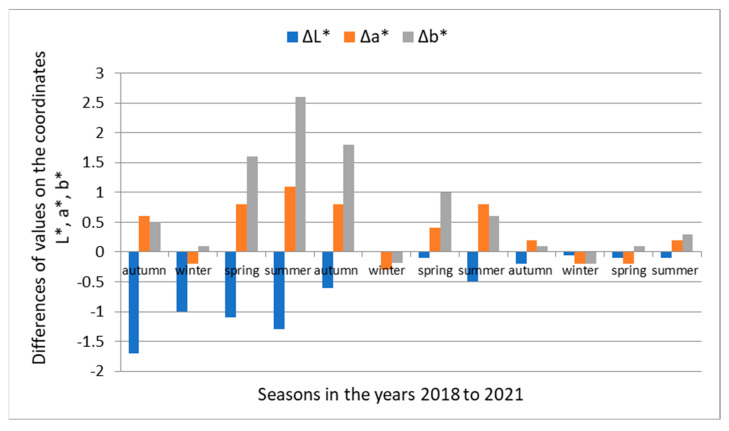
Magnitudes of changes in the ∆L*, ∆a*, ∆b* values in the color space CIE L*a*b* native beech wood during the 36-month exposure to sunlight, depending on the season.

**Figure 6:**
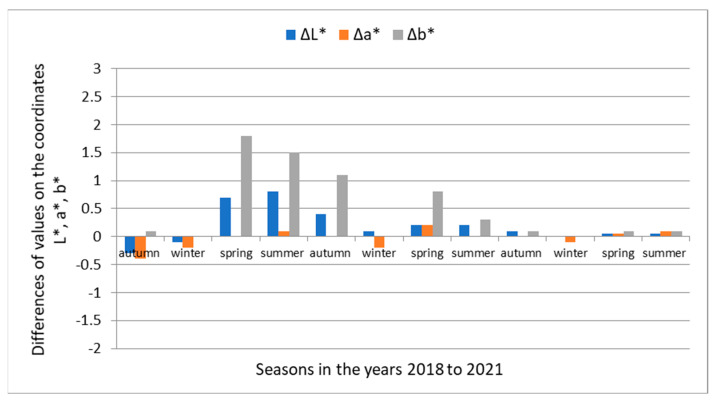
The magnitudes of changes in the values of ∆L*, ∆a*, ∆b* in the color space CIE L*a*b* of steamed beech wood during the 36-month exposure to sunlight, depending on the season.

The course of the measured values on the light coordinate L*, the chromatic coordinates of color a*, and the yellow color b* in Figure 3 and Figure 4 during 36 months of dazzling is not fluent. The fluctuations are attributed to the influence of the intensity of solar radiation during the individual seasons, causing photolytic and photo-oxidative reactions of daylight radiation with wood. Figure 5 and Figure 6 show the magnitudes of changes in the average values of ∆L*, ∆a*, ∆b* in individual seasons during the exposure.

**Figure 7:**
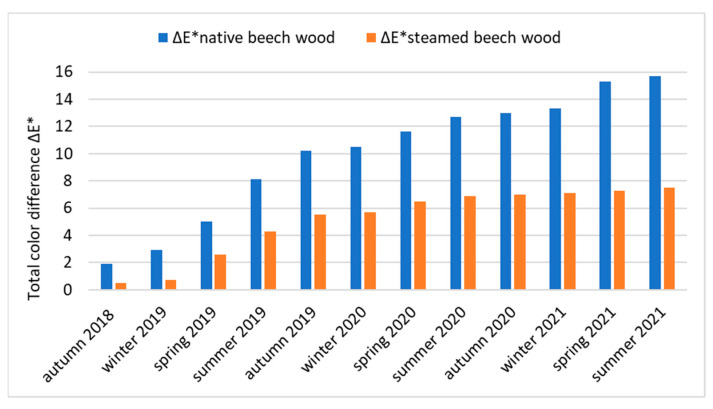
Values of the total color difference ∆E* of native and steamed beech wood during 36 months of dazzling (October 2019 to October 2021).

In Figure 7, the degree of the color change of the surface of native and steamed beech wood caused by solar radiation during 36 months is documented by the total color difference ∆E*.

From the comparison of wood colors in Figure 2 and the values presented at the coordinates L*, a*, b* of unsteamed and steamed beech wood during the exposure, Figure 3 and Figure 4 show that while the surface of the unsteamed beech wood darkened and browned, the brown-red color on the steamed wood lightened.

The darkening and browning of unsteamed beech wood numerically documents the shift of the brightness coordinate L* from the value *L*_0_* = 76.6 to *L*_36_* = 71.3, i.e., by the value ∆L* = −5.3, and changes in chromatic coordinates: red color a* from *a*_0_* = 7.8 to *a*_36_* = 12.2, i.e., by the value ∆a* = +4.4, and the yellow color b* from the value *b*_0_* = 19.8 to *b*_36_* = 26.8, i.e., by the value ∆b* = +10.0. The largest darkening of unsteamed beech wood occurred during the first year of dazzling, when changes in the brightness coordinate ΔL* reached 76.9% of the total change in brightness of beech wood caused by daylight; in the second year, it reached 16.8 and in the third year 6.3%. The browning of unsteamed beech wood is described by changes in the chromatic coordinates: red a* and yellow b*. The change in the red coordinate in the first year of exposure was 57.5% of the total change ∆a*, in the second year of dazzling 42.5% and in the third year oscillated around a* = 12. In the yellow coordinate, the change ∆b* in the first year of dazzling was 57.7% of the total value of the change in ∆b* beech wood, 38.7% in the second year and 3.6% in the third year. The changes in the red a* and yellow b* coordinates of the color space CIE L*a*b* in the third year, as the measurements show, are small, and, in addition, the different seasons are contradictory, while in winter and spring, they show a decrease in values, so in summer, in times of more intense sunlight, they grow. The darkening of wood due to solar radiation is in line with the views of experts dealing with changes in the properties of wood due to long-term exposure to sunlight, who state that the wood surface darkens and mostly yellows and browns [2,3,8,19,20].

Steamed beech wood under the influence of sunlight for 36 months compared to unsteamed wood showed the opposite character of the color change, where the surface of the wood faded. Visually, this is documented in Figure 2, as well as the shift of the brightness coordinate L* from the value *L*_0_* = 55.3 to *L*_36_* = 57.2, i.e., by the value ∆L* = +1.9, on the red coordinate a* offset from *a*_0_* = 14.5 to *a*_36_* = 14.1, i.e., by the value ∆a* = −0.4, and on the chromatic coordinate of yellow color b* from the value *b*_0_* = 19.8 to *b*_36_* = 25.5, i.e., by the value ∆b* = +5.7. On the basis of comparison of individual changes ∆L*, ∆a*, ∆b* on the coordinates of the color space CIE L*a*b* of steamed beech wood caused by the action of sunlight with changes ∆L*, ∆a*, ∆b* on the coordinates of the unmatched beech wood caused by daylight, it can be stated that the values expressing the magnitude of changes in steamed beech wood are smaller. The magnitude of changes in the brightness coordinates L* and yellow b*, similar to unsteamed beech wood, is largest in the first year of exposure. The red coordinate changes of a* oscillated around the value of a* = 14.0. In winter, at low sunlight intensity, the values on the red coordinate a* decreased, and from spring to autumn, they increased at higher sunlight intensity. The rate of decline or the increase in red coordinate values decreases over the years. Based on the above findings, it can be stated that the functional groups of chromophores in beech wood with absorption of the electromagnetic radiation spectrum with a 630–750 nm red wavelength causing reddening of steamed beech wood were steamed and strongly eliminated for photochemical reactions of wood caused by daylight.

The authors of [21,22] point out the effect of lightening the surface of steamed wood under the action of UV radiation. In the work, a team of authors [21] report the lightening of the surface color of steamed maple wood after its irradiation in Xenotest with a 450 xenon lamp emitting UV radiation with a wavelength of 340 nm, 42 ± 2 W/m^2^ intensity, for 7 days. The lightening of the red-brown color of steamed maple wood is declared by the increase in the values on the brightness coordinate from *L*_1_* = 65.3 to the value of *L*_2_* = 70.7, i.e., by the value ∆L* = +5.4, the increase in the value on the chromatic coordinate of the yellow color from *b*_1_* = 19.4 to the value *b*_2_* = 28.9, i.e., by the value ∆b* = +9.3, with a slight change in the red coordinate value from *a*_1_* = 10.8 to *a*_2_* = 10.3, i.e., by the value ∆a* = −0.5.

The influence of UV radiation on steamed acacia wood in [22] states that while the surface of steamed acacia wood darkened slightly at the steaming temperature *t* = 100 °C, the surface of acacia wood lightened at the steaming temperature *t* = 120 °C.

The authors [23] describe the positive effect of the steaming process on the decomposition of functional groups of maple wood chromophores manifested by the darkening and browning of maple wood and the elimination of photochemical reactions caused by daylight. They point to the fact that the greater the darkening of the maple wood in the steaming process, the smaller the color changes on the surface of the irradiated steamed maple wood by UV radiation. This is declared by the decrease in the total color difference from the value ∆E* = 18.5 for unsteamed maple wood to ∆E* = 7.2 and steamed maple wood with saturated water steam with temperature *t* = 135 °C, as well as the results of FTIR analyses.

The contribution of the influence of beech wood steaming on the color fastness and resistance to the effects of sunlight declares a decrease in the value of the total color difference ∆E* in Figure 7. While the change in the color of unsteamed beech wood caused by solar radiation expressed by the value of the total color difference over 3 years is ∆E* = 15.7, the change in the total color difference of steamed beech wood in the same period is ∆E* = 7.5, which is a decrease in color change by about 52.2%. This points to the fact that beech wood steaming has a positive effect on changes in the chromophore system of steamed beech wood and the partial resistance of steamed beech wood to the initiation of photochemical reactions induced by daylight wavelengths.

**Figure 8:**
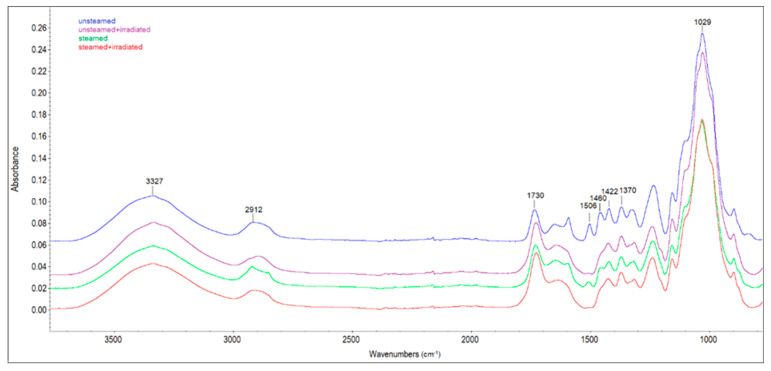
FTIR spectra of beech wood samples.

**Figure 9:**
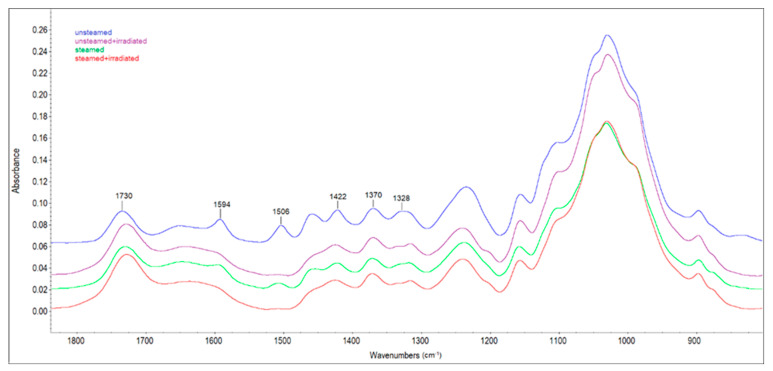
FTIR spectra of beech wood samples in the range 1800 to 800 cm^−1^.

Chemical changes on the surface of unsteamed and steamed beech wood before and after solar irradiation were also monitored by ATR-FTIR spectroscopy. The FTIR spectra of the examined samples in Figure 8 show the whole range of wavenumbers 4000 to 650 cm^−1^. In Figure 9, only spectra in the range 1800 to 800 cm^−1^ are presented, where most of the specific vibrations occurred.

During various thermal treatments of wood, the chemical composition changes in the wood, which depends on the experimental treatment conditions, such as the temperature, time and atmosphere used. However, many competitive reactions take place simultaneously, depending on experimental conditions. For these reasons, our results may differ from those of other authors.

The bands in the range of 3800 to 2750 cm^−1^ are assigned to hydroxyl and methyl/methylene stretching vibrations [24]. In the FTIR spectra at 1730 cm^−1^ (assigned to unconjugated carbonyl groups), the increase in absorbance, between the original beech wood before and after irradiation and the steamed beech wood before and after irradiation, was due to an increase in carbonyl or carboxyl groups in lignin or carbohydrates (Figure 9). At this observed band, the highest increase in absorbance (by 34%) was recorded in the spectrum of the irradiated steamed beech wood sample. The results of the FTIR analysis also suggest that the lignin macromolecule changes after sun irradiation of the samples by reducing the absorption bands characteristic of lignin: 1594 cm^−1^ (belonging to aromatic skeletal vibration in lignin, and -C=O stretching [25]), 1506 cm^−1^ (C=C stretching of the aromatic skeletal vibrations in lignin [26]), 1422 cm^−1^ (assigned to aromatic skeletal vibrations combined with C-H deformation in carbohydrates [27]). The absorption band at 1506 cm^−1^ almost disappeared with the irradiated steamed sample, and the reduction of the band intensity was by more than 93% compared to the steamed sample. The reduction of band intensity for lignin in wood samples after UV irradiation was also observed by the authors [28]. In our research, we also recorded a decrease in absorbances at bands 1421 cm^−1^ (aromatic ring vibration in lignin combined with C−H deformation in carbohydrates) and 1328 cm^−1^ (C-O vibration in syringyl plus guaiacyl derivatives is characteristic for condensed structures in lignin) when comparing unsteamed beech wood samples before and after irradiation, as well as steamed wood and irradiated steamed beech wood. This decrease indicates cleavage of the methoxyl groups during sunlight, leading to gradual demethoxylation. The reduction in the intensity of the bands at 1124 cm^−1^ (C-H vibration of syringyl units in lignin) irradiated wood samples indicates cleavage of the ether bond in the lignin structure.

The intensity of the band at 1370 cm^−1^ (C-H deformations in polysaccharides) is not significantly affected by the treatment conditions of the wood samples, so this band was used as a reference to determine lignin degradation. To compare the changes in chemical composition on the surface of the wood samples used for the experiment, the ratios of intensities 1506/1370, 1730/1370 and 1730/1506 were calculated (Table 3).

The decrease in the 1506/1370 ratio of intensities in the wood samples after solar irradiation demonstrates the decay of lignin, which occurs due to the interaction of radiation with wood. The increase in unconjugated carbonyl groups (carbonyl-containing chromophores) due to photo-oxidation is reflected in the increasing ratio of 1730/1370 (carbonyl/carbohydrates). These results are consistent with published research by other authors [29,30,31]. The relative increase in carbonyl groups in lignin or carbohydrates with the relative decrease in lignin in parallel is reflected in an increase in the 1730/1506 ratio. The authors of [32] explained the increase in this ratio by primarily lignin degradation and oxidation (photo-oxidation and thermo-oxidation resulting in an increase in carbonyl groups).

The authors of [33] used FTIR analysis of chemical changes in beech and oak wood after steaming and compression. Using FTIR, they observed changes in the hydroxyl groups, as well as in the C-O and C-H functional groups in the polysaccharides and in lignin on the surface of the samples. Other authors [34,35,36,37,38] state that the lightfastness of various woody plants to a large extent mainly affects lignin and extractives.

## 4. Conclusions

This paper presents the results of the color change of the surface of unsteamed and steamed beech wood with saturated water steam in a pressure autoclave, which was exposed to daylight interior for 36 months. The results of analyses of the effect of solar radiation on unsteamed and steamed beech wood showed:

1. The color surface of unsteamed beech wood changed under the influence of daylight during the exposure. The wood darkened and browned into a brown-yellow color shade. The opposite tendency, i.e., lightening, occurred during exposure on steamed beech wood samples.
2. The measured changes in the values at the coordinates of the color space CIE L*a*b* caused by solar radiation in unsteamed beech wood are: ΔL* = −5.3; Δa* = +4.4; Δb* = +10.0. Changes were recorded also for steamed beech wood: ΔL* = +1.9; Δa* = -0.4; Δb* = 5.7.
3. The evaluation of the color change of beech wood in the form of the total color difference ΔE* shows that the surface of steamed beech wood shows approximately 52.2% less color change than the total color difference of unsteamed beech wood.
4. The decrease in the values of ΔL*, Δa*, and Δb* and the total color difference ΔE* of steamed beech wood indicate a positive effect of steaming of wood on the partial resistance of steamed beech wood to the initiation of photochemical reactions induced by wavelengths daylight.
5. The lightfastness of wood is greatly affected by lignin. The main change in the ATR-FTIR spectra is the decrease in lignin absorption bands in parallel with the increase in unconjugated carbonyl absorption after irradiation of unsteamed and steamed beech wood with daylight components. Photodegradation of irradiated unsteamed and steamed wood was associated with a reduction in the ratio of lignin to carbohydrates while at the same time increasing the ratio of unconjugated carbonyl to carbohydrates, which may affect the partial resistance of steamed beech wood to daylight.

## Figures and Tables

**Table 1:** Mode of color modification of beech wood with saturated water steam.

Mode	Temperature of Saturated Water Steam (°C)			Time of Operation (h)		
	*t_min_*	*t_max_*	*t*_4_	τ_1_-Phase I	τ_2_-Phase II	Total Time
Mode	132.5	137.5	100	6.0	1.0	7.5

**Table 2:** Coordinate values of color space CIE L*a*b* of unsteamed and steamed beech wood.

Beech Wood	Unsteamed Beech Wood			Steamed Beech Wood		
	Coordinates of Color Space CIE L*a*b*					
	L*	a*	b*	L*	a*	b*
Before exposure	76.6 ± 1.7	7.8 ± 1.5	19.8 ± 1.7	55.3 ± 1.6	14.5 ± 1.0	23.5 ± 0.8
After exposure	71.3 ± 0.9	12.2 ± 0.6	26.8 ± 0.9	57.6 ± 0.9	14.1 ± 0.8	27.5 ± 0.6

**Table 3:** Ratios of absorption bands for the beech wood.

Assignment	Ratio of Intensities	Unsteamed	Unsteamed + Irradiated	Steamed	Steamed + Irradiated
Nonconjugated carbonyl/carbohydrates	1730/1370	2.04	2.72	2.49	3.19
Lignin/carbohydrates	1506/1370	1.03	0.08	0.43	0.03
Nonconjugated carbonyl/lignin	1730/1506	1.98	33.40	5.78	119.00
